# WSES guidelines updates

**DOI:** 10.1186/s13017-020-00318-z

**Published:** 2020-06-10

**Authors:** Marco Ceresoli, Federico Coccolini, Walter L. Biffl, Massimo Sartelli, Luca Ansaloni, Ernest E. Moore, Salomone Di Saverio, Yoram Kluger, Fausto Catena

**Affiliations:** 1 General and Emergency Surgery Department, School of Medicine and Surgery, Milano-Bicocca University, Monza, Italy; 2 General, Emergency and Trauma Surgery Department, Pisa University Hospital, Pisa, Italy; 3 Trauma Surgery Department, Scripps Memorial Hospital La Jolla, San Diego, CA USA; 4 General and Emergency Surgery, Macerata Hospital, Macerata, Italy; 5 General, Emergency and Trauma Surgery Department, Bufalini Hospital, Cesena, Italy; 6 Trauma Surgery, Denver Health, Denver, CO USA; 7 Department of General Surgery, University Hospital of Varese, University of Insubria, Varese, Italy; 8 Division of General Surgery, Rambam Health Care Center, Haifa, Israel; 9 Emergency and Trauma Surgery, Maggiore Hospital, Parma, Italy

**Keywords:** Guidelines, Evidence-based medicine, Guideline development, Guideline update, WSES

## Abstract

The World Society of Emergency Surgery promotes training and continuing medical education in the field of emergency surgery and trauma. One of the most important activities of the society is the development of guidelines. The debate about the process of developing and updating guidelines is very active with no clear consensus and different policies among scientific societies. The present commentary provides the position of the World Society of Emergency Surgery on guideline development process and their update.

## Background

The World Society of Emergency Surgery (WSES) was established in 2007 with the aim to promote training and continuing medical education in emergency general surgery and trauma.

Since then, great effort was made in that direction: six world congresses with vast international attendance took place and several international educational meetings were initiated as well as numerous educational courses in different fields of trauma and emergency surgery were conducted and above all, WSES launched and curated several clinical guidelines for emergency and trauma surgery [1, 2].

Some guidelines have already been updated, and others will be updated in the near future.

## Main text

The debate about the clinical guideline updating process is very animated with many scientific societies facing this problem. No consensus on update timing is acceptable by all [3, 4].

No clear agreement exists about the timing and methodology for guideline updates. Some authors advocate updates every 2–5 years, and some advocate an update only when relevant evidences are available or recommendations become outdated [5–7].

### Generally strong recommendations last longer

#### Are our recommendations really strong?

The relationship between emergency surgery and evidence-based medicine unfortunately at the moment is a troubled story. Good-quality evidence is frequently lacking in emergency surgery, and the difficulty to conduct randomized trials in emergency settings is well documented [8–10].

In this context, the quality of existing evidences is poor and based mainly on retrospective and observational studies; therefore, the strength of recommendations is often weak, based more on the plausibility that even a well-designed study will not change the clinical practice rather than on the results of randomized trials.

Moreover, the development of clinical guidelines is an extremely complex process that involves laborious work and involvement of an international panel of experts and needs months of work of reviewing the literature, mediating several points of view, and experts’ opinions. In the absence of solid and strong evidences, like in emergency surgery, this process becomes harder.

At the light of these considerations, and in order to avoid a waste of time in producing duplicate guidelines with similar recommendations, the WSES board has decided to adopt a new policy and recommendations.

- WSES requires a good-quality standard for guidelines; the development of guidelines should be performed according to the AGREE II requirements adopting the GRADE methodology for evidence evaluation and grading [11, 12].
- For each guideline topic, WSES will appoint a committee with a coordinator that will be nominated by WSES board who will be in charge of the continuous monitoring of new evidences available about the topic
- The nominated committee will be responsible to add, on a dedicated area in the WSES website, all the relevant updates and new studies. The committee will have to notify and propose to the WSES board the need for guideline updating.

WSES will continue to promote a scientific approach to emergency and trauma surgery and to support the advance of clinical studies such the International Register of Open Abdomen (IROA), the International Register of Emergency Surgery (WIRES), and the ongoing COOL study [13, 14] in order to obtain good-quality evidence and the development of a better quality evidence-based approach in the daily practice.

## Conclusion

WSES guidelines will be developed according to high-quality standards; a dedicated committee, nominated by the WSES board, will be responsible for the continuous evaluation of new evidences. The development of an updated version of guidelines will be promoted and evaluated by the WSES board in case of important changes and new evidences available.

## Data Availability

Not applicable

## Abbreviations

IROA: International Register of Open Abdomen
WIRES: WSES International Register of Emergency Surgery
WSES: World Society of Emergency Surgery
